# Supplementary material for: Comparative validation of the BOADICEA and Tyrer-Cuzick breast cancer risk models incorporating classical risk factors and polygenic risk in a population-based prospective cohort of women of European ancestry
Source: Breast Cancer Res. 2021 Feb 15;23:22. doi: 10.1186/s13058-021-01399-7 (PMC7885342; doi:10.1186/s13058-021-01399-7)
Supplement: Supplementary file 1 — Additional file 1: Supplementary Materials: Comparative validation of the BOADICEA and Tyrer- Cuzick breast cancer risk models incorporating classical risk factors and polygenic risk in a population-based prospective cohort of women of European ancestry.Additional details on the definition of study follow-up, sources of genotype data, risk factors in BOADICEA and Tyrer-Cuzick models, model validation methods are given. Supplementary Fig. 1. shows the study design in the validation cohort. Supplementary Fig. 2. shows a comparative validation of Tyrer-Cuzick model with and without PRS in the Generations Study. Supplementary Table 1 shows the risk factor distribution in the Generation Study. Figure 1. Calibration and discrimination of five-year risk predictions of breast cancer for women younger than 50 years in the nested case-control sample of the Generations Study cohort with risk categories based on deciles of predicted five-year absolute risk. Figure 2. Calibration and discrimination of five-year risk predictions of breast cancer for women aged 50 years or older in the nested case-control sample of the Generations Study cohort with risk categories based on deciles of predicted five-year absolute risk. [file 13058_2021_1399_MOESM1_ESM.docx]

**Supplementary Materials: Comparative validation of the BOADICEA and Tyrer-Cuzick breast cancer risk models incorporating classical risk factors and polygenic risk in a population-based prospective cohort of women of European ancestry**

**Definition of follow-up and sources of genotype data**

The follow-up of these subjects were defined from the date of study entry to the date of the latest of the two follow-up questionnaires, for details we refer to earlier papers (1,2).

The genotyping and construction of the 313-variant polygenic risk score (PRS) are described in Mavaddat *et al.* (3).

**Risk factors in extended BOADICEA and IBIS models**

The incorporation of reproductive and lifestyle risk factors and the 313-SNP PRS to the original BOADICEA model (version 5.0) are described in detail in Lee *et al.* (4) (see Table 2 of Lee *et al.* for a summary). For details on the risk factors included in the IBIS (version 8.0) model we refer the reader to Pal Choudhury *et al.* (2), in particular Supplementary Table 4 of that paper. The 313-SNP PRS is incorporated to this model using the approach described in Brentnall *et al.* (5)*.* The characteristics of the reproductive, lifestyle factors and PRS for the women in the validation study are shown in Supplementary Table 1. To deal with missing risk factor data, the BOADICEA model estimates risk for each woman using only the observed risk factor information (4), and the IBIS model assigns a population risk (corresponding to relative risk 1.00) (6).

**Model validation methods**

The standardized model validation methods recently implemented in the iCARE tool (7) were used to run comparative validation analyses of five-year absolute risk estimated based on the updated versions of the BOADICEA and Tyrer-Cuzick models. Further details on methods are provided in Pal Choudhury *et al.* (7). In brief, the models predicting five-year risk of breast cancer were evaluated for calibration, i.e., whether the model-based risks are unbiased for subjects with different risk factor profiles and discrimination, i.e., the ability of the model to separate the cases and controls. The follow-up for a subject was defined to be the minimum of observed follow-up and five years. Absolute risk calibration was evaluated by comparing the observed number of breast cancer patients within the follow-up period and the expected number of such patients estimated by the model, both overall and within deciles of predicted absolute risk accounting for multiple comparisons for the ten decile categories using Bonferroni correction. The calibration slope and intercepts were reported based on a linear regression of the decile-specific observed proportion of cases within five-years and average of the predicted five-year absolute risk. Calibration was also assessed in terms of relative risks, defined with respect to the average five-year risk in the study population, through a similar comparison of the observed and expected quantities. Model discrimination was assessed using the Area Under the Receiver Operating Characteristics Curve (AUC). We account for the non-random sampling of cases and controls using sampling weights by an inverse probability weighted approach (7).

**Supplementary Figure 1:** Study design for the validation cohort

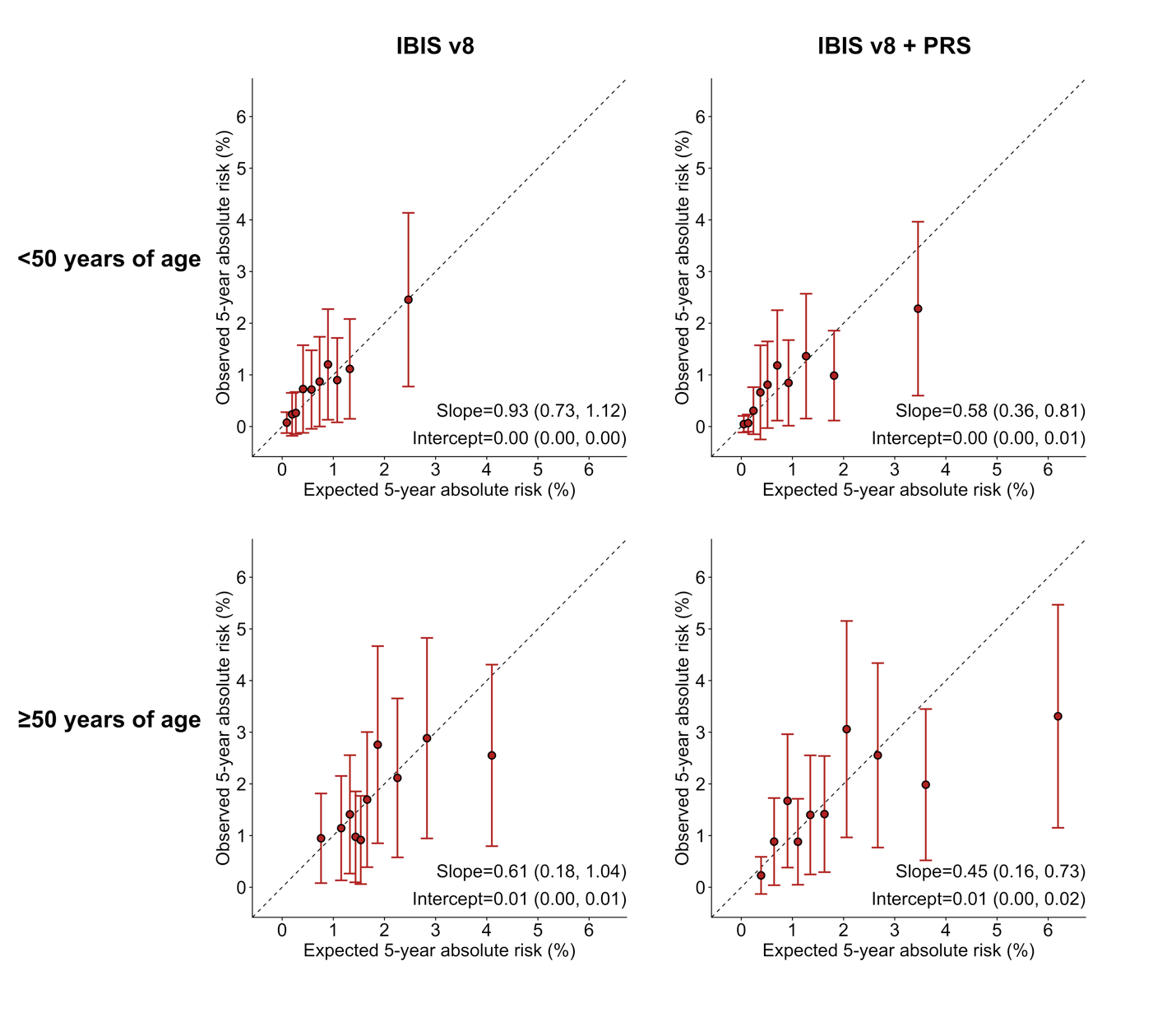
**Supplementary Figure 2:** Calibration and discrimination of five-year absolute risk predictions of breast cancer based on the Tyrer-Cuzick model before [IBIS v8] and after [IBIS v8 + PRS] incorporating the recently developed PRS, based on 313 common germline variants, for women in the nested case-control sample of the Generations Study cohort with risk categories based on deciles of predicted five-year absolute risk. Estimates and 95% confidence intervals of calibration slope and intercept are also reported.

IBIS = International Breast Cancer Intervention Study, PRS = polygenic risk score

**Supplementary Table 1.** Risk factor distributions in the Generations Study*

| **Breast cancer risk factor** | **Ages <50** | | | | **Ages ≥50** | | | |
| --- | --- | --- | --- | --- | --- | --- | --- | --- |
|  | **Non-Cases, N=233** | | **Cases, N=207** | | **Non-Cases, N=485** | | **Cases, N=412** | |
| **Age at baseline, years** | |  |  |  |  |  |  |  |
| Median (range) | 44 | (23-49) | 44 | (24-49) | 59 | (50-75) | 59 | (50-75) |
| **PRS** |  |  |  |  |  |  |  |  |
| Median (range) | -0.30 | (-2.67-1.52) | -0.17 | (-1.88-1.34) | -0.40 | (-2.21-1.42) | -0.20 | (-1.67-1.61) |
| **Age at menarche, years** | |  |  |  |  |  |  |  |
| ≤11 | 54 | (26.5) | 36 | (19.1) | 88 | (20.6) | 84 | (22.3) |
| 12-13 | 102 | (50.0) | 98 | (52.1) | 214 | (50.1) | 189 | (50.3) |
| 14-15 | 42 | (20.6) | 48 | (25.5) | 111 | (26.0) | 93 | (24.7) |
| ≥16 | 6 | (2.9) | 6 | (3.2) | 14 | (3.3) | 10 | (2.7) |
| Missing | 29 |  | 19 |  | 58 |  | 36 |  |
| **Parity** |  |  |  |  |  |  |  |  |
| Nulliparous | 56 | (24.0) | 46 | (22.2) | 63 | (13.0) | 51 | (12.4) |
| 1 birth | 33 | (14.2) | 26 | (12.6) | 54 | (11.1) | 42 | (10.2) |
| 2 births | 108 | (46.4) | 102 | (49.3) | 247 | (50.9) | 218 | (52.9) |
| 3+ births | 36 | (15.5) | 33 | (15.9) | 121 | (24.9) | 101 | (24.5) |
| Missing | 0 |  | 0 |  | 0 |  | 0 |  |
| **Age at first birth, years (among parous women)** | | | |  |  |  |  |  |
| <20 | 7 | (4.0) | 8 | (5.0) | 17 | (4.0) | 19 | (5.3) |
| 20-24 | 26 | (14.7) | 30 | (18.6) | 162 | (38.4) | 114 | (31.6) |
| 25-29 | 71 | (40.1) | 67 | (41.6) | 169 | (40.0) | 148 | (41.0) |
| ≥30 | 73 | (41.2) | 56 | (34.8) | 74 | (17.5) | 80 | (22.2) |
| Missing | 0 |  | 0 |  | 0 |  | 0 |  |
| **OC use** |  |  |  |  |  |  |  |  |
| Never | - | | - | | 98 | (20.3) | 89 | (21.6) |
| Ever |  |  |  |  | 385 | (79.7) | 323 | (78.4) |
| Missing |  |  |  |  | 2 |  | 0 |  |
| **Current OC use (among women age <50)** | | | |  |  |  |  |  |
| Never | 6 | (2.6) | 13 | (6.3) | - | | - | |
| Former | 198 | (85.0) | 154 | (74.4) |  |  |  |  |
| Current | 29 | (12.4) | 40 | (19.3) |  |  |  |  |
| Missing | 0 |  | 0 |  |  |  |  |  |
| **HRT use (among women age ≥50)** | | |  |  |  |  |  |  |
| Never | - | | - | | 236 | (49.0) | 196 | (47.8) |
| Former |  |  |  |  | 161 | (33.4) | 119 | (29.0) |
| Current |  |  |  |  | 85 | (17.6) | 95 | (23.2) |
| Missing |  |  |  |  | 3 |  | 2 |  |
| **Type of HRT use (among current users age ≥50)** | | | |  |  |  |  |  |
| Current E-type | - | | - | | 43 | (56.6) | 41 | (45.6) |
| Current C-type |  |  |  |  | 33 | (43.4) | 49 | (54.4) |
| Missing |  |  |  |  | 9 |  | 5 |  |
| **Age at menopause, years (among women age ≥50)** | | | |  |  |  |  |  |
| <40 | - | | - | | 7 | (2.5) | 3 | (1.3) |
| 40-44 |  |  |  |  | 16 | (5.7) | 17 | (7.4) |
| 45-49 |  |  |  |  | 61 | (21.9) | 53 | (22.9) |
| 50-54 |  |  |  |  | 159 | (57.0) | 127 | (55.0) |
| ≥55 |  |  |  |  | 36 | (12.9) | 31 | (13.4) |
| Missing |  |  |  |  | 206 |  | 181 |  |
| **Height, m** |  |  |  |  |  |  |  |  |
| Median (range) | 1.7 | (1.5-1.8) | 1.7 | (1.4-1.8) | 1.6 | (1.4-1.9) | 1.6 | (1.4-1.8) |
| **Body mass index, kg/m^2^** | |  |  |  |  |  |  |  |
| <25 | 110 | (48.7) | 124 | (61.7) | 250 | (52.3) | 192 | (47.3) |
| ≥25 - <30 | 80 | (35.4) | 46 | (22.9) | 159 | (33.3) | 147 | (36.2) |
| ≥30 | 36 | (15.9) | 31 | (15.4) | 69 | (14.4) | 67 | (16.5) |
| Missing | 7 |  | 6 |  | 7 |  | 6 |  |
| **Alcohol, g/day** |  |  |  |  |  |  |  |  |
| None | 33 | (14.2) | 31 | (15.0) | 90 | (18.9) | 83 | (20.5) |
| <5 | 20 | (8.6) | 24 | (11.6) | 61 | (12.8) | 46 | (11.4) |
| 5-14 | 72 | (30.9) | 61 | (29.5) | 126 | (26.5) | 107 | (26.4) |
| 15-24 | 47 | (20.2) | 43 | (20.8) | 104 | (21.8) | 76 | (18.8) |
| 25-34 | 35 | (15.0) | 28 | (13.5) | 53 | (11.1) | 49 | (12.1) |
| 35-44 | 15 | (6.4) | 12 | (5.8) | 25 | (5.3) | 19 | (4.7) |
| ≥45 | 11 | (4.7) | 8 | (3.9) | 17 | (3.6) | 25 | (6.2) |
| Missing | 0 |  | 0 |  | 9 |  | 7 |  |
| **History of BBD** |  |  |  |  |  |  |  |  |
| No | 193 | (82.8) | 153 | (73.9) | 346 | (71.3) | 270 | (65.5) |
| Yes | 40 | (17.2) | 54 | (26.1) | 139 | (28.7) | 142 | (34.5) |
| Missing | 0 |  | 0 |  | 0 |  | 0 |  |
| **Breast cancer family history in first degree relatives** | | | | |  |  |  |  |
| No | 190 | (81.5) | 144 | (69.6) | 382 | (78.8) | 301 | (73.1) |
| Yes | 43 | (18.5) | 63 | (30.4) | 103 | (21.2) | 111 | (26.9) |
| Missing | 0 |  | 0 |  | 0 |  | 0 |  |
| * Risk factor distributions are reported as n (%), unless otherwise specified. BMI and alcohol intake were assessed at baseline. OC use was specified as never/ever for women 50 years or older and never/former/current for women younger than 50 years; in each case the distributions are only reported for the corresponding age group. HRT use, HRT type use and age at menopause were only included in the model for women 50 years or older and the distributions are shown for the older group only, BBD = benign breast disease, C-type = estrogen and progestogen combined, E-type = estrogen-only, HRT = hormone replacement therapy, OC = oral contraceptive. | | | | | | | | |

**References**

1. Swerdlow AJ, Jones ME, Schoemaker MJ, et al. The Breakthrough Generations Study: Design of a long-term UK cohort study to investigate breast cancer aetiology. *Br J Cancer*. 2011. doi:10.1038/bjc.2011.337

2. Choudhury PP, Wilcox AN, Brook MN, et al. Comparative validation of breast cancer risk prediction models and projections for future risk stratification. *JNCI J Natl Cancer Inst*. 2019. doi:10.1093/jnci/djz113

3. Mavaddat N, Michailidou K, Dennis J, et al. Polygenic Risk Scores for Prediction of Breast Cancer and Breast Cancer Subtypes. *Am J Hum Genet*. 2019. doi:10.1016/j.ajhg.2018.11.002

4. Lee A, Mavaddat N, Wilcox AN, et al. BOADICEA: a comprehensive breast cancer risk prediction model incorporating genetic and nongenetic risk factors. *Genet Med*. 2019. doi:10.1038/s41436-018-0406-9

5. Brentnall AR, van Veen EM, Harkness EF, et al. A case-control evaluation of 143 single nucleotide polymorphisms for breast cancer risk stratification with classical factors and mammographic density. *Int J Cancer*. 2019. doi:10.1002/ijc.32541

6. Brentnall AR, Cuzick J, Buist DSM, Bowles EJA. Long-Term accuracy of breast cancer risk assessment combining classic risk factors and breast density. *JAMA Oncol*. 2018. doi:10.1001/jamaoncol.2018.0174

7. Pal Choudhury P, Maas P, Wilcox A, et al. iCARE: An R package to build, validate and apply absolute risk models. *PLoS One*. 2020;15(2):e0228198. https://doi.org/10.1371/journal.pone.0228198.
